# Pathogenesis of Focal Segmental Glomerulosclerosis and Related Disorders

**DOI:** 10.1146/annurev-pathol-051220-092001

**Published:** 2025-01

**Authors:** Mehmet M. Altintas, Shivangi Agarwal, Yashwanth Sudhini, Ke Zhu, Changli Wei, Jochen Reiser

**Affiliations:** 1 Department of Internal Medicine, University of Texas Medical Branch, Galveston, Texas, USA;; 2 Grove Biopharma, Inc., Chicago, Illinois, USA; 3 Department of Internal Medicine, Rush University Medical Center, Chicago, Illinois, USA

**Keywords:** kidney, podocyte, innate immunity, integrin, FSGS, suPAR

## Abstract

Focal segmental glomerulosclerosis (FSGS) is the morphologic manifestation of a spectrum of kidney diseases that primarily impact podocytes, cells that create the filtration barrier of the glomerulus. As its name implies, only parts of the kidney and glomeruli are affected, and only a portion of the affected glomerulus may be sclerosed. Although the diagnosis is based primarily on microscopic features, patient stratification relies on clinical data such as proteinuria and etiological criteria. FSGS affects both children and adults and has an elevated risk of progression to end-stage renal disease. The prevalence of FSGS is rising among various populations, and the efficacy of various therapies is limited. Therefore, understanding the pathophysiology of FSGS and developing targeted therapies to address the complex needs of FSGS patients are topics of great interest that are currently being studied across various clinical trials. We discuss the etiology of FSGS, describe the major contributing pathophysiological pathways, and outline emerging therapeutic strategies along with their pitfalls.

## 1. INTRODUCTION

The kidney is a complex organ harboring more than 30 different cell types, some of them highly specialized. The classification and diagnosis of focal segmental glomerulosclerosis (FSGS) lie within this complexity, largely manifesting with a highly variable clinical course. The origin of the injury can be traced back only by evaluating the structural pattern of injured renal cells, which are mainly podocytes (1) but also parietal epithelial cells (PECs), glomerular endothelial cells, and, later, tubular epithelial cells (2). Tracking the progression of FSGS requires a thorough analysis of a heterogeneous group of pathogenic events including glomerular scar formation (capillary collapse, podocyte hypertrophy and hyperplasia), extracellular matrix (ECM) accumulation, and glomerular basement membrane (GBM) thickening (3). Clinically, patients with FSGS presenting with asymptomatic proteinuria may develop nephrotic-range proteinuria and glomerulosclerosis with kidney function decline as long-term outcomes (4), while urinary protein reduction for preserving kidney function in FSGS has been proven beneficial (5). FSGS patients can experience swelling and uremia which indicate advanced disease with a poor prognosis (6). They have increased risk of developing cardiovascular complications, progressing to end-stage renal disease (ESRD) (which may be treated by dialysis or transplantation) or premature death. Unfortunately, current treatments do not adequately address the complex challenges of FSGS, which accounts for 40% of glomerular kidney disease cases in adults and 20% in children (7) and is a leading cause of kidney failure globally (8).

FSGS should not be considered as a single disease but rather as a histological pattern of (biopsy-diagnosed) glomerular scars (9). It presents with patchy involvement that can ultimately result in severe complications such as permanent kidney damage and loss of kidney function, especially if left untreated. FSGS can be classified as primary (idiopathic), secondary (including viral infection, drugs, postinflammatory conditions, or vascular issues), or genetic on the basis of the integration of phenotypic and genotypic profiles. These categorizations have recently been refined by KDIGO (Kidney Disease: Improving Global Outcomes) in its clinical practice guidelines (10). In the absence of a genetic or an identifiable secondary cause, FSGS with diffuse foot process (FP) effacement is typically caused by permeability factor(s) (11) and has been categorized as permeability factor–related FSGS (12). For those without diffuse FP effacement, FSGS is classified in a distinct group called FSGS of undetermined cause (10).

**Figure 1:**
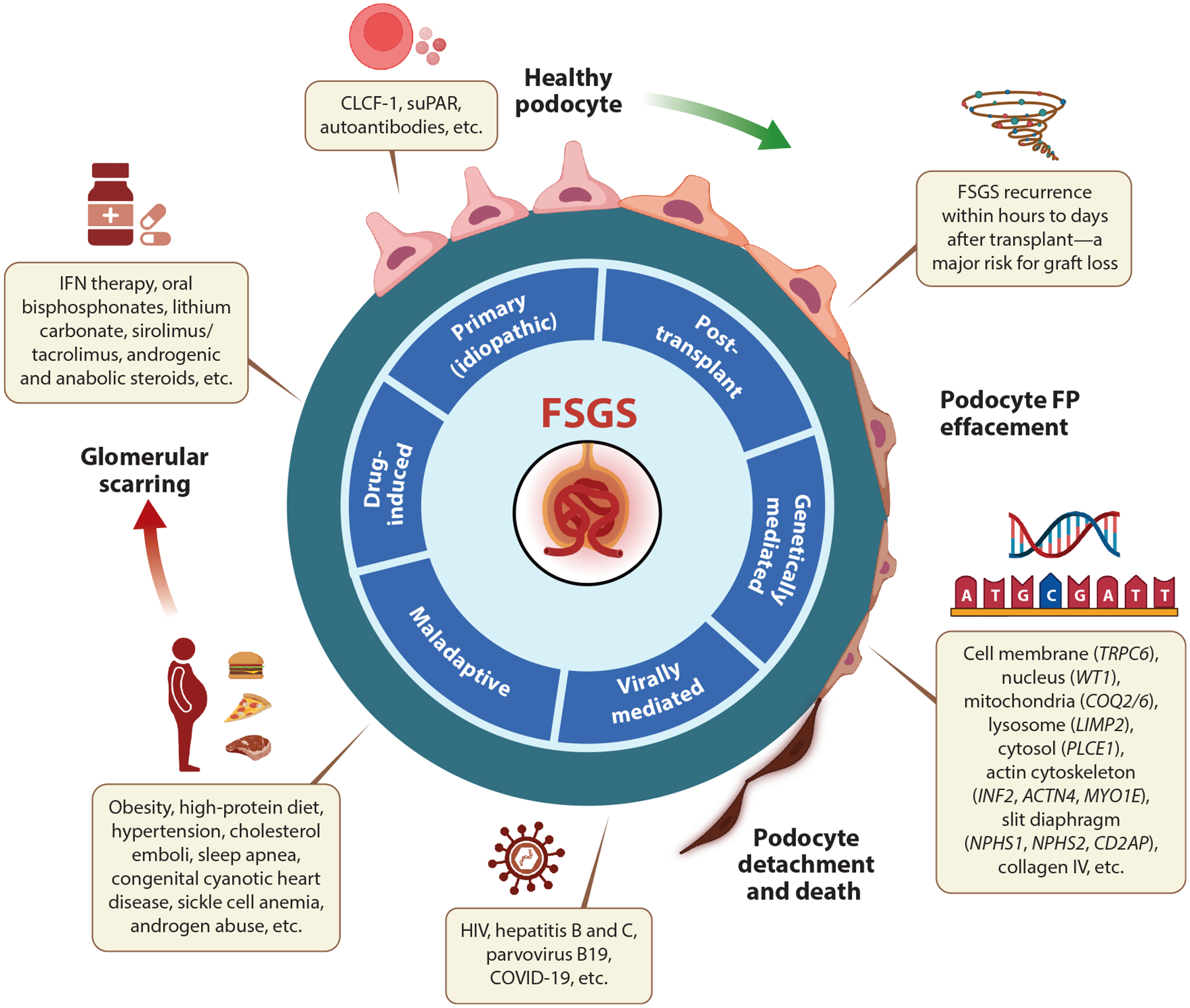
The spectrum of glomerular injury leading to the disruption of the glomerular filtration barrier in FSGS. Abbreviations: COVID-19, coronavirus disease 2019; FP, foot process; FSGS, focal segmental glomerulosclerosis; HIV, human immunodeficiency virus; IFN, interferon; suPAR, soluble urokinase-type plasminogen activator receptor. Figure adapted from images created with BioRender.com.

In this review, we focus on the mechanisms of glomerular (especially podocyte) injury causing FSGS (Figure 1) and discuss new insights that could facilitate development of novel therapies aimed at slowing down, stopping, or reversing the progression of FSGS.

## 2. PRIMARY FSGS

The most common cause of primary FSGS is generalized podocyte injury (or podocytopathy), which is often induced by a circulating factor that can cross the GBM barrier and signal to podocytes. This factor can originate from outside the kidney. However, in most cases, the condition develops rapidly and suddenly on its own without an obvious or a known cause. Primary FSGS is thus diagnosed when no secondary or genetic cause can be identified.

### 2.1. Circulating Permeability Factors

From the initial observation by Gentili et al. (13) in 1954 to Shalhoub’s (14) hypothesis in 1974 to our current understanding, knowledge of the role of circulating permeability factor(s) in primary FSGS has come a long way. The main indications that suggest the existence of pathogenic permeability factor(s) in primary FSGS are as follows. (*a*) Some patients with primary FSGS developed recurrence after transplantation (15–17). While proteinuria could be present within hours to days after kidney transplantation (18), FP effacement could be detected even within minutes after reperfusion (19). (*b*) Two independent case reports showed that when retransplanted into a non-FSGS patient, the allograft suffering from recurrent FSGS (rFSGS) regained function in a new host without history of FSGS and rFSGS resolved (20, 21). (*c*) A baby girl delivered from her FSGS mother presented with nephrotic range proteinuria, which was resolved spontaneously within four days after birth, suggesting that the glomerular permeability factor was transmitted from the mother to her child (22). (*d*) Plasmapheresis and/or immunoadsorption reduced proteinuria in patients with rFSGS (23, 24). (*e*) Serum or plasma from active FSGS patients could induce proteinuria in experimental animals (25–27) and cause an increased glomerular permeability to albumin in perfused rat glomeruli in vitro (28).

The identification of permeability factors has been challenging. Over the last few decades, different candidates were proposed and studied to various degrees. Out of all of them, soluble urokinase-type plasminogen activator receptor (suPAR) has been studied the most, as it fulfills the criteria for a circulating factor: It is produced outside the kidney, it travels in the bloodstream, it signals to podocytes causing proteinuria, its removal or blockade alleviates podocyte injury and proteinuria, and its reintroduction causes proteinuria. Circulating factors with more or less published evidence are (*a*) cytokines: cardiotrophin-like cytokine factor 1 (CLCF-1) and tumor necrosis factor-α (TNF-α) (29–31); (*b*) protease or protease-related proteins: suPAR, calcium/calmodulin-serine protease kinase (CASK) (32), metalloproteinase-2 (MMP-2), and tissue inhibitor of metalloproteinase-1 (TIMP-1) (33); (*c*) autoantibodies: anti-CD40, anti–angiotensin II type 1 receptor (AT1R) (34, 35), and anti-nephrin; (*d*) microRNAs (miRNAs): miR-30c (36), miR-125b, and miR-192 (37); and (*e*) others: CD40L (CD154) (38).

Next, we focus on some of the commonly discussed circulating factors.

#### 2.1.1. CLCF-1.

To facilitate the study of permeability factors, Savin et al. (28) established an in vitro permeability assay, in which isolated rat glomeruli were subjected to FSGS plasma or serum. Compared with normal subjects, rFSGS sera induced an enhanced glomerular permeability to albumin. The group identified a protein <30 kDa from the permeability active fraction of FSGS samples, which was later identified as CLCF-1 (39, 40). Further, the glomerular permeability was blocked by a CLCF-1 monoclonal antibody (40). CLCF-1 belongs to the interleukin 6 (IL-6) family and shares structural similarity with cardiotrophin-1 and ciliary neurotrophic factor. It is expressed in a variety of immune and nonimmune tissues and has been implicated in different cellular events (41). While CLCF-1 has been shown to be increased in some rFSGS (41), it has never been verified independently in any large cohort, leaving its clinical implication still unknown. Similarly, its effect on podocyte pathobiology is not clear either, though CLCF-1 treatment caused changes in actin cytoskeleton in cultured murine podocytes (41).

#### 2.1.2. suPAR.

suPAR is by far the most studied and documented circulating factor that is both a risk factor and a biomarker of kidney disease (42). It is the soluble form of uPAR, a three-domain glycosylphosphatidylinositol (GPI)-anchored protein that binds the protease uPA, facilitating the generation of activated plasmin. In addition to its role in directional invasion of migrating cells, uPAR also functions as a signaling molecule via its interaction with α_v_β_3_ integrin on podocytes (43). Initial study of suPAR in renal diseases focused on FSGS. Findings indicated that it was particularly increased in primary and rFSGS patients (44). This observation was confirmed in a follow-up study with two independent FSGS cohorts in which suPAR was elevated in two-thirds of the patients (45). Additionally, suPAR could be removed by plasmapheresis in some posttransplant FSGS patients, which was associated with improved clinical outcome (44, 46). Experimentally, suPAR activated podocyte α_v_β_3_ integrin in both native and grafted kidneys; overexpression of suPAR caused FP effacement, proteinuria, and FSGS-like glomerulopathy in mice (44). Interestingly, bone marrow–derived immature myeloid cells were identified as the main source of renal pathogenic suPAR (47). Together, these studies concluded that suPAR meets the criteria of a glomerular permeability factor.

The identification of suPAR as a glomerular permeability factor has prompted numerous preclinical and clinical investigations. Circulating suPAR levels do not differentiate primary FSGS from secondary FSGS or from other glomerular disease, indicating that suPAR is not specific to primary FSGS (48–50). Indeed, the elevation of suPAR, as an immune factor, has been observed in various clinical conditions, including systemic inflammation and malignant diseases, conditions that can present with proteinuria (51). Various large-cohort studies demonstrated that baseline suPAR levels could independently predict the incidence of chronic kidney disease (CKD) and progression to loss of kidney function, as indicated by an overall decline of estimated glomerular filtration rate (eGFR) (52). Considering that a high level of suPAR is also a risk factor for acute kidney injury (AKI) in different clinical and experimental settings (53, 54), it is reasonable to consider circulating suPAR as a general kidney disease risk factor and biomarker in clinical practice.

In addition to clinical studies with suPAR, there have been discrepancies in terms of the effect of suPAR in experimental models. An increase of suPAR levels via injection of recombinant protein or genetic overexpression from liver does not cause proteinuria in mice within hours (55, 56), but proteinuria is seen with longer exposure or expression from other tissue sources (57, 58). The discrepancy could be attributed to the individual experimental design and setting (59), but the complexity of suPAR and uPAR could play a role as well, especially because they exist in different transcriptional isoforms and enzymatic cleavage fragments (60). To further understand this phenomenon, our group created three transgenic mouse models: overexpressing full-length mouse suPAR, secreted/short form of mouse suPAR (lacking a GPI anchor and a part of C-terminal D2D3 domains), and mouse suPAR D2D3 fragment. Interestingly, overexpression of different forms of mouse suPAR generated different renal phenotypes in mice. The secreted/short form of mouse suPAR generated spontaneous proteinuria and developed an FSGS-like nephropathy in mice fed high-fat diet, while full-length mouse suPAR and D2D3 fragment required high-fat diet to develop proteinuria (47, 61).

#### 2.1.3. CD40 autoantibodies.

CD40 belongs to the TNF receptor superfamily. It is a transmembrane protein typically present on B cells, dendritic cells, and macrophages, but it can be detected on some nonhematopoietic cells, such as podocytes (62). When exploring potential biomarkers for prediction of FSGS recurrence, Delville et al. (62) identified a panel of seven autoantibodies that could predict posttransplant FSGS recurrence with up to 92% accuracy in their cohort. Among the panel, elevation of pretransplant CD40 autoantibody had the single best correlation with FSGS recurrence after transplantation (62). While injection of the CD40 autoantibody purified from rFSGS patients alone did not per se cause proteinuria in mice, co-treatment of both CD40 autoantibody and suPAR triggered FP effacement and proteinuria, suggesting the synergy between these two circulating factors in causing glomerular disease (62, 63). In a follow-up study, FSGS serum with high levels of both CD40 autoantibody and suPAR was able to induce podocyte injury, which could be inhibited by either anti-uPAR or anti-CD40 antibodies (64).

#### 2.1.4. Anti-nephrin autoantibodies.

Nephrin is an indispensable podocyte slit diaphragm (SD) protein that is important for glomerular ultrafiltration (65); mutations in nephrin cause the congenital nephrotic syndrome of the Finnish type (66). Watts et al. (67) identified the presence of anti-nephrin autoantibody in 29% of patients with biopsy-proven minimal change disease (MCD). Along with the previously published animal studies indicating that anti-nephrin antibody could induce proteinuria, they proposed the autoimmune mechanism responsible for some MCD (67). Interestingly, a multi-institutional pediatric cohort study in Japan demonstrated a possible implication of anti-nephrin autoantibody in posttransplant FSGS recurrence (68). The authors showed that the plasma level of anti-nephrin was high in rFSGS patients before transplantation or during recurrence, whereas the anti-nephrin level in nonrecurrent FSGS patients and genetic FSGS patients was comparable with that of control subjects (68). Although this study was limited by size and did not interrogate pretransplant samples in all patients, it set the tone for how the anti-nephrin autoantibody was involved in rFSGS as a circulating factor. In a recent retrospective multicenter study on rFSGS, Batal et al. (69) found elevated pretransplant anti-nephrin in some but not all patients with proven rFSGS, but none in adult patients without recurrence. Their findings suggested that anti-nephrin antibodies before transplantation were associated with posttransplant recurrence but negative anti-nephrin did not exclude recurrence. A most recent study by Hengel et al. (70) identified anti-nephrin antibodies as a reliable biomarker for tracking disease progression in kidney disorders with nephrotic syndrome.

In summary, there is clear clinical and experimental evidence indicating the role of circulating factor(s) in primary FSGS. While new candidates may be discovered, a one-size-fits-all FSGS factor is unlikely, reflecting the heterogeneity of primary FSGS. Given the findings that suPAR synergizes with CD40 autoantibody or apolipoprotein L1 (*APOL1*) risk variants to cause proteinuria (62, 71), it is possible that multiple circulating factors work together to trigger primary FSGS or its recurrence after transplantation.

### 2.2. Clinical Features and Pathogenesis

Primary FSGS is a heterogeneous group of clinical and pathological entities that are characterized by the full nephrotic syndrome, including nephrotic-range (>3.5 g/day protein) proteinuria [or even massive (>10 g/day) protein loss in the urine (72)] as well as reduced plasma albumin concentrations (hypoalbuminemia; <30 g/L) without the presence of genetic or secondary cause (10). These clinical presentations are often associated with other medical conditions such as renal insufficiency, hematuria, and hypertension (73) in both children and adults. Histological manifestation of primary FSGS is portrayed by the effacement of podocyte FPs (74) in at least one glomerulus (75), leading to denudation of the GBM and attachment of a podocyte-denuded capillary to Bowman’s capsule, as depicted by Kriz et al. (76, 77) for the representation of the progression through segmental to global sclerosis.

The pathophysiology of primary FSGS is still poorly understood such that it is unclear why sclerotic lesions are found in part of the glomeruli even though all podocytes are likely affected by and vulnerable to complications. Another feature of FSGS kidney biopsies is the pathological heterogeneity; that is, samples often contain scarred and histologically normal glomeruli side by side. This complicates the interpretation and initial diagnosis of FSGS. Furthermore, FP effacement is extensive in primary FSGS and is even observed along the nonsclerosed glomeruli (78, 79). These imply that identification of the diverse glomerular lesions solely by light microscopy underestimates the number of sclerotic lesions, and it is certainly not sufficient for an accurate assignment to the appropriate diagnosis and management. Besides, qualitative content of manual light microscopy analysis depends on the user to a greater or lesser extent, and this interobserver variability is a serious limitation. Therefore, electron microscopy (EM) of renal biopsy is often needed and is required to detect early structural changes of FSGS. To eliminate the possibility of missing the diagnosis due to the focal distribution of FSGS lesions, Fuiano et al. (80) studied the distribution and size of sclerotic lesions by performing three-dimensional morphometric analysis using 182 glomeruli from 14 biopsies from patients with primary FSGS. Their findings suggested that (*a*) focal sclerotic lesions affected only 12.5% of the total glomerular volume, (*b*) the distribution of sclerotic lesions in primary FSGS is not focal, but rather is virtually diffuse, and (*c*) precise quantification of sclerotic glomeruli requires the analysis of at least 10 glomeruli obtained from both cortical and juxtamedullary zones [since juxtamedullary nephrons may be affected first (81)] per biopsy. Hence, renal biopsy and three-dimensional morphometric analysis of the entire glomerulus are crucial for identification of primary FSGS and precise quantification of sclerotic glomeruli, respectively.

In another study using computer-assisted morphometric analysis of endothelial cell injury in primary FSGS, researchers examined the alteration of glomerular capillary networks in biopsies from 29 cases diagnosed with primary FSGS and 18 controls (82). Their analysis showed that FSGS glomeruli had a smaller capillary lumen area but a larger ECM area when compared with the healthy glomeruli, indicating diffuse and global endothelial damage and dysfunction in FSGS glomeruli as well as altered communication between podocytes and endothelial cells (83). In-depth investigations of FSGS etiology at the omics level are also emerging. These studies aim to quantify mRNA expression of several genes as well as map the exact location of gene activity within the kidney using spatial transcriptomic profiling methods. In one study including 38 FSGS patients and 10 healthy controls, several small RNAs (e.g., miR-21–5p and miR-192–5p) were found to be differentially expressed according to the histological origin of the lesions in the primary FSGS (84).

PECs were also implicated in the pathogenesis of primary FSGS. The development of segmental sclerotic lesions starts with severe podocyte damage and detachment, which are followed by PEC activation and migration from Bowman’s capsule to replace podocyte loss to some extent and restore glomerular filtration (84–86). To further validate this phenomenon, a study by Sakamoto et al. (87) traced the podocyte lineage and showed that the direction of phenotypic transition in primary FSGS was from podocytes to PECs.

### 2.3. Recurrence of FSGS Posttransplantation

FSGS is known to frequently recur after kidney transplant and is associated with poor allograft survival in up to 60% of cases (88–90). FSGS recurrence can be sudden, with the appearance of nephrotic-range proteinuria within the first few days or even hours after transplantation (91). The reported rates of recurrence are quite variable, from 4% to 66%, depending on the population characteristics under study (92, 93).

Of note, patients who had lost their allograft to rFSGS are usually not retransplanted since the risk of recurrence in the subsequent allograft is increased by ~80–100%. However, not all patients with FSGS encounter recurrence after kidney transplant. Like genetic FSGS, secondary FSGS has a negligible or low risk of recurrence. Therefore, pretransplant counseling regarding recurrence is critical, and patients with FSGS must undergo pretransplant genetic screening. Pretransplant treatment using plasmapheresis has been shown to have preventive effects on FSGS recurrence after kidney transplant (94).

The suggested risk factors for recurrence include age at onset of disease, rapid progression to ESRD (<48–72 months), history of previous recurrence in an allograft, and higher proteinuria levels pretransplantation (93, 95, 96). It is noteworthy that there have been controversial reports on age being a risk factor. Although younger patients are considered to be at higher risk than older patients (97), other studies have reported higher risks in adults than in children (98), with another group concluding that there is no difference between adults and children (99). These differing and opposing results could be attributed to the small sample size of the study populations in most of these reports. An interesting study by Ding et al. (100) showed that children with steroid-resistant nephrotic syndrome (SRNS) who initially responded to steroid treatment were at a higher risk of recurrence after kidney transplant. A recent review by Troyanov et al. (101) describes in detail the risk factors for FSGS relapse.

## 3. SECONDARY FSGS

While primary FSGS occurs without an identifiable cause, secondary FSGS is associated with several etiologies, including various malignancies, infection, and drugs, as outlined below.

### 3.1. Drug-Induced FSGS

Drug-associated FSGS is defined as podocytopathies that are induced in response to therapeutic drugs, causing iatrogenic forms of glomerular diseases (102). Multiple therapeutic agents or commonly prescribed medications such as interferons (IFN-α, -β and -γ), bisphosphonates (pamidronate), lithium, sirolimus, and anabolic steroids are implicated in inducing two types of FSGS: FSGS not otherwise specified (NOS) and collapsing FSGS (103). In most cases, drug-induced FSGS can revert, and renal function recovers upon discontinuation of the drugs and treatment with glucocorticoids (104).

IFNs are indicated for the treatment of viral infections, multiple sclerosis, malignant osteoporosis, and many other medical conditions; for instance, while IFN-α is administered for treatment of viral infections such as hepatitis B and C, IFN-β is extensively used for multiple sclerosis, and IFN-γ is used for chronic granulomatous disease and malignant osteopetrosis. Since podocytes express receptors for both IFN-α and -β and express a major histocompatibility complex (MHC) class II antigen in response to IFN-γ, all forms of IFNs could induce FSGS via a direct effect on podocytes (105, 106). Interestingly, cases of collapsing FSGS after treatment with IFN were predominant in the Black population. DNA analysis from six Black patients and one Hispanic patient revealed them to be homozygous for high-risk *APOL1* alleles, and IFN was shown to increase *APOL1* expression, at least in vitro (107). The study points toward the possibility that IFN production, as a part of innate response to viral infection, may play a role in inducing renal disease in patients with high-risk *APOL1* alleles and that treatment with exogenous IFN may exacerbate the disease by acting as a second hit.

Unlike oral bisphosphonates, high dosage of intravenously administered nitrogen containing pamidronate, used to treat metastatic bone disease, hypercalcemia of malignancy, and multiple myeloma, had detrimental effects on the podocyte cytoskeleton and impaired cellular energetics, but the exact mechanism for how it causes nephrotoxicity (collapsing FSGS) is unknown (108). Pamidronate-associated collapsing FSGS results in florid nephrotic syndrome and renal insufficiency with poor outcomes, and in one study only one-half of the cases showed improvement in renal function after drug discontinuation, with none returning to baseline (109).

Lithium carbonate, widely used to treat bipolar disorders, is associated with adverse renal effects including nephrogenic diabetes insipidus, MCD, AKI, and acute tubular necrosis (ATN) (110). While association of lithium with FSGS is nebulous, there have been case reports of patients who developed FSGS after a lithium regime (111–113).

Use of sirolimus is accompanied by subnephrotic proteinuria in transplant patients (114) and by induction of de novo FSGS as well (115). In vitro studies have demonstrated that exposure to sirolimus caused a reduction in synaptopodin, nephrin, and podocin expression; increased apoptosis; and actin cytoskeletal reorganization in podocytes, explaining the potential basis for its cytotoxicity (116).

Long-term androgenic anabolic steroid abuse by bodybuilders and weight lifters has also been associated with the development of both NOS and collapsing FSGS; weaning from the steroid use showed a reduction in proteinuria and stabilization of overall renal function (117). Moreover, FSGS associated with severe tubulointerstitial lesions was also reported in patients taking cocaine and heroin (118).

Fairly recently, development of nephrotic syndrome was observed shortly after the start of guselkumab for the treatment of plaque psoriasis; however, it was resolved after cessation of the drug without relapse (119). Interestingly, a few other cases of checkpoint inhibitor–induced FSGS have been reported in patients receiving cancer therapies (120). Substance abuse (heroin, cocaine, nicotine, and alcohol) and recreational drugs are also being investigated for their potential nephrotoxic effects (121).

### 3.2. Maladaptive FSGS

Maladaptive FSGS is caused by a reduction in the number of functional nephrons (unilateral renal agenesis, renal dysplasia, oligomeganephronia–congenital nephropenia, or low nephron endowment at birth) or when a normal nephron population encounters an abnormal hemodynamic stress resulting in dysfunctional reparative processes and focal sclerosis (morbid obesity, surgical reduction of renal mass by >75%, reflux nephropathy, high-protein diet, or sickle cell disease) (103). Other causes include but are not limited to sleep apnea, cyanotic congenital heart disease, renal artery stenosis, malignant hypertension, and cholesterol emboli. These conditions lead to a mismatch between glomerular load and glomerular capacity, wherein the former exceeds the latter, putting an excessive workload on the nephron (122). Consequently, an increased glomerular load associated with hyperfiltration induces an untenable mechanical and rheological stress on podocytes. To redistribute the mechanical forces and decrease the local shear stress, podocytes start to efface but only in some of the afflicted segments (123). In a kidney biopsy, maladaptive FSGS presents as large glomeruli, a preponderance of perihilar scars in glomeruli with sclerotic changes, and only a partial FP effacement. Unlike primary FSGS, where the disease onset is rapid and abrupt with global and extensive damage to podocytes, in secondary or adaptive forms of FSGS, the insult is slow and sustained; therefore, the disease progression, manifestation of clinical symptoms, and FP effacement are relatively mild and only segmental. Patients with maladaptive FSGS develop subnephrotic- or nephrotic-range proteinuria but rarely exhibit nephrotic syndrome. Reducing injurious glomerular capillary hypertension, typically with renin-angiotensin-aldosterone system (RAAS) inhibitors, is the main line of treatment for maladaptive FSGS, whereas glucocorticoids and other immunosuppressive drugs generally remain ineffective (8, 124).

### 3.3. Infection-Related FSGS

FSGS has been associated with various causes, including viral infections such as human immunodeficiency virus (HIV), hepatitis B and C, and parvovirus B19 (PVB19) (125). Virus-related FSGS is also a significant factor in the development of collapsing FSGS, which is characterized by the segmental or global condensation and obliteration of glomerular capillaries, along with the emergence of hyperplastic and hypertrophic podocytes and severe tubulointerstitial damage (126). The pathomechanism of viral nephropathy varies depending on the specific virus and the type of glomerular disease.

#### 3.3.1. HIV-related FSGS.

HIV-associated nephropathy (HIVAN) exhibits a distinctive histology, manifesting as a collapsing variant of FSGS. The pathogenesis of HIVAN involves localized HIV infection of the kidney, with the virus targeting both tubular and glomerular epithelial cells. While FSGS is the primary glomerular lesion in HIVAN, other documented glomerular pathologies in HIV patients encompass IgA nephropathy, cryoglobulinemia, amyloidosis, and a lupus-like immune complex glomerulopathy (127).

A mounting body of evidence suggests that a mutation in the *APOL1* gene is necessary for the onset of HIVAN, meaning the viral immune response of HIV in concert with *APOL1* risk protein. Among the various variants of the *APOL1* gene, the G1 and G2 genetic variants are notable. These variants are associated with CKD, FSGS, and HIVAN (128).

#### 3.3.2. Hepatitis B virus–related and hepatitis C virus–related FSGS.

Globally, there are an estimated 257 million individuals infected with the hepatitis B virus (HBV) (129). Reports indicate that chronic HBV infections are linked to various forms of glomerulopathies, including HBV-associated FSGS in adults (130). The pathogenesis of HBV-induced FSGS is elusive. Sakai and colleagues (130) identified HBV DNA in urinary podocytes of a patient with chronic HBV infection and the collapsing variant of FSGS, suggesting that podocytes may serve as a potential direct target for the virus.

Case reports on hepatitis C virus (HCV)-related FSGS are scarce. Hogan et al. (131) documented three cases of patients with HCV infection, solid organ transplantation, and initially normal kidney function who developed proteinuria while undergoing treatment with direct-acting antiviral therapies. Subsequent kidney biopsies revealed FSGS without evidence of immune complex or cryoglobulinemic glomerulonephritis.

#### 3.3.3. Parvovirus B19–related FSGS.

PVB19-associated glomerulonephritis typically manifests in the second or third decade and exhibits histological patterns consistent with mesangiocapillary glomerulonephritis or endocapillary glomerulonephritis. However, it is worth noting the existence of a collapsing variant of FSGS, which may also be linked to PVB19. This collapsing FSGS resembles the lesions observed in HIVAN, yet these individuals are HIV negative (132). While there is no direct evidence linking the infection to glomerulonephritis, the heightened presence of PVB19 DNA in individuals with idiopathic or collapsing FSGS suggests a potential pathogenic involvement of PVB19 in renal disease development, though causality remains unproven to date (133).

#### 3.3.4. New cases of collapsing FSGS.

Renal involvement, characterized by AKI, proteinuria, and hematuria, has been frequently observed in coronavirus disease 2019 (COVID-19) patients, exacerbating the overall prognosis (134). Biopsy and autopsy studies have revealed that the majority of COVID-19-associated AKI is attributed to ATN. However, glomerular involvement has also been documented and warrants distinction from the typical ATN-related AKI cases (135).

COVID-19 can trigger a cytokine storm, resulted in an uncontrolled release of cytokines, which will further induce podocyte damage and collapsing FSGS (136, 137). The circulating factors might be involved in this process. Recently, Wei et al. (138) reported a novel observation regarding a distinct form of proteinuria in patients infected with non-omicron variants that step-wisely correlates with elevated suPAR levels. Mechanistically, their findings suggest that the SARS-CoV-2 (severe acute respiratory syndrome coronavirus 2) spike S1 protein and suPAR synergistically activate α_v_β_3_ integrin, which contributes to podocyte injury.

## 4. MORPHOLOGICAL CLASSIFICATION AND DIFFERENTIATION OF FSGS

### 4.1. Morphological Variants of Primary and Secondary FSGS

Sclerotic lesions of primary and secondary FSGS represent a morphologic spectrum of immunohistochemically characterized glomerular regions. Morphological classification of FSGS, also referred to as Columbia classification, is introduced to identify these lesions on the basis of light microscopic examination (139). According to this classification, FSGS lesions are categorized into five patterns: collapsing variant, tip variant, cellular variant, perihilar variant, and FSGS-NOS (Table 1).

This grading system is predictive of prognosis as well as outcomes and is validated to be useful in the management of disease. In a multivariate analysis of 225 FSGS patients, Stokes and colleagues (145) found that collapsing, cellular, and tip lesion variants displayed significantly different rates of remission (13.2%, 44.5%, and 75.8%, respectively), which correlated with the renal survival outcomes in these variants, that is, the worst for the collapsing variant and the best for the tip lesion variant. Another retrospective study with a similar follow-up period that consisted of 197 patients also concluded that collapsing FSGS had the lowest remission (18%) and 3-year renal survival rates (33%), whereas tip lesion FSGS had the highest remission (53%) and 3-year renal survival rates (76%) (146). D’Agati et al. (147) reported that there were significant differences in demographic features and clinical presentations between FSGS variants. Also, the collapsing variant had the strongest association with Black people (63%), whereas the tip variant had the strongest association with White people (86%) and the best baseline variables compared with other variants (147).

### 4.2. Differentiating Primary from Secondary FSGS

The distinction between primary and secondary FSGS is sometimes not recognized clearly, and the morphologic variants may not solely help to distinguish between these two diverse forms. Rather, those variants tend to be correlated with certain clinical features.

Further clinicopathologic studies have clearly and elegantly made the distinction between the primary and secondary forms of FSGS. In one study, the FP width measured by EM was used as an independent factor to differentiate primary and secondary FSGS: the median FP widths in primary (*n* = 17) and secondary (*n* = 7) FSGS were measured as 3,236 and 1,098 nm, respectively, as compared with 562 nm in controls (*n* = 12) (148). The authors concluded that an FP width of 1,500 nm is the threshold value, above which primary FSGS is differentiated from secondary FSGS with high sensitivity and specificity. Sethi and colleagues (149) defined primary FSGS as having nephrotic syndrome, extensive (80% to 100%) FP effacement on electron micrographs of kidney biopsies, and the absence of risk factors associated with secondary FSGS. Non-nephrotic syndrome–associated FSGS with segmental (20% to 60%) FP effacement measured by EM potently suggests otherwise, i.e., a secondary FSGS (149). The same group later clarified a few key points in a follow-up study. They pointed out that a relatively intact and nonsclerotic glomerulus must be selected to achieve a consistent ultrastructural analysis; otherwise, the analysis would reveal a widespread FP effacement in the setting of either primary or secondary FSGS (150). A small note here is that, even though the FP effacement is relatively mild in secondary forms of FSGS due to the slow and sustained insult, toxic drug-induced (105) and virus-induced (151) secondary FSGS are exceptions, and these can lead to extensive FP effacement.

A recent study shows promise for using a defined set of urinary peptide biomarkers as a non-invasive approach to selectively discriminate primary from secondary FSGS with high sensitivity and specificity (152).

When FSGS cannot be classified by any of the above-mentioned approaches or techniques, genetic analysis should be performed to unveil any undiagnosed genetic basis for FSGS (79).

## 5. GENETIC CAUSES OF FSGS

Although FSGS is considered a podocyte disease, its etiology remains diverse and heterogeneous (1, 153). In addition to other factors, genetic mutations have been attributed to driving FSGS (154). In individuals who present with SRNS, a causative genetic variant has been identified in 10% of adult-onset FSGS, and this rate is as high as 43% in familial cases (155). Interestingly, genetic defects have been identified in up to two-thirds (~60%) of patients with FSGS, manifesting in the first year of their lives (156). However, a direct causal relationship between the genetic mutation and disease progression (proteinuria and renal failure) is not so obvious in older children and adults with FSGS, suggesting that an additional second hit (genetic or environmental) may be required.

The genetic causes of FSGS may present as sporadic or familial disease with autosomal dominant (AD), autosomal recessive (AR), X-linked, or mitochondrial (matrilineal) inheritance patterns. As a rule of thumb, most of the dominant forms of FSGS are caused by gain-of-function mutations and are characterized by later disease onset with gradual progression to renal failure. On the other hand, the recessive forms of FSGS, which are more aggressive than the AD forms, occur because of loss-of-function mutations and manifest in infancy or early childhood. FSGS-causing mutations (~50 genes have been discovered so far) are generally localized in genes that encode proteins that are essential for the assembly and maintenance of podocyte ultrastructure (including actin cytoskeleton and SD) and function (157). For example, mutations occur in proteins localized to the cell membrane (*TRPC6*), nucleus (*WT1*), mitochondria (*COQ2* and *COQ6*), lysosomes (*LIMP2*), and cytosol (*PLCE1*). Other common mutations are found in genes encoding actin cytoskeletal (*INF2*, *ACTN4*, *MYO1E*) and SD (*NPHS1*, *NPHS2*, *CD2AP*) proteins. Furthermore, mutations in the structural GBM glycoproteins of the collagen IV lineage (*COL4A3*/*A4*/*A5*) simply induce a cytoskeletal disorganization that makes the podocyte more vulnerable. Such genetic alterations have also been recognized as more common causes of FSGS (158). Interestingly, FSGS may also ensue from mutations in genes that do not exclusively encode for podocyte-specific proteins; rather, these proteins are present more abundantly in other tissues or cell types. In such syndromic forms of FSGS, the extrarenal manifestations are more prominent and often diagnostic. There have been other studies that primarily focus on genetic causes of FSGS, which may be either limited to the kidney or part of a broader syndrome with extrarenal involvement (79, 159–161).

Interestingly, sickle cell disease (SCD), an AR monogenetic chronic anemia syndrome caused by a point mutation in the β-globin gene, has been associated with kidney injury referred to as sickle cell nephropathy (SCN) that progresses to ESRD (162). Patients with SCN quickly advance through the stages of tubular dysfunction and hyperfiltration, heavy proteinuria, and loss of GFR. With the loss of other nephrons, single-nephron GFR increases, causing progressive damage to the glomeruli, which manifests as FSGS and interstitial fibrosis along with tubular atrophy.

While there are no clear-cut clinical or histopathological parameters for distinguishing genetic FSGS from other types, there are several hallmarks of genetic disease such as family history, onset of disease at an early age, and atypical severity along with steroid resistance (79, 157). As per the definition, genetic FSGS does not recur after kidney transplantation; however, rare and exceptional cases of “recurrent” proteinuria have been described in patients with mutations in the nephrin (*NPHS1*) gene, which were due to the development of anti-nephrin antibodies following kidney transplantation (163). Intriguingly, a study described the degree of FP effacement as varied in patients with mutations in the genes encoding proteins associated with SD and cytoskeletal proteins, while the degree of FP effacement in those with mutations in other FSGS genes was described as segmental. Evaluating the degree of FP effacement using EM thus could serve as a useful tool to differentiate between primary and secondary (genetic) FSGS (164), as explained above. With the recent technological advancements, genetic testing using next-generation sequencing of either the entire genome (whole-genome sequencing), protein-coding regions (whole-exome sequencing), or a specific set of genes of interest (targeted gene panel) for detecting and diagnosing disease-causing genes in genetic FSGS has become commonplace in clinical practice (165, 166).

Racial and ethnic backgrounds are likely to have a substantial influence on the frequency, distribution, incidence, and progression of FSGS in children and adults. Considering a significant racial and ethnic predilection, the incidence rates for FSGS in adults are approximately 1.5- to 2-fold higher in men than in women. The incidence of FSGS is approximately 5 times higher in Black patients when compared with White patients in the United States (167, 168), which could be attributed to an increased frequency of polymorphism/variation in two risk/susceptibility loci of nonmuscle myosin heavy chain-9 (*MYH9*) (169) and *APOL1* (170, 171). In a cohort of 205 African American patients with biopsy-proven FSGS, Genovese et al. (172) showed that the *APOL1* G1 haplotype was present in 52% of FSGS cases as opposed to 18% in controls. Similarly, a case-control study by Kopp et al. (173) reported that individuals with two G1 risk alleles had 17 times increased odds for the development of FSGS. However, the pathophysiology behind this association remained elusive for several years until our group showed the formation of an offensive tripartite complex between suPAR, *APOL1* risk variants, and α_v_β_3_ integrin inflicting damage to podocytes (71). Geographical connections have also been observed; for instance, mutations in nephrin (*NPHS1*) are the rule (>95%) in Finland, whereas mutations in podocin (*NPHS2*) are the dominant cause of genetic FSGS in other European countries (174). On the other hand, *NPHS2* mutation is rare in Korean, Chinese, and Japanese populations (175). To obtain further in-depth insight into the correlation between genetic FSGS and ethnicity/race in children and adults, the reader is referred to other review articles (160, 167).

## 6. CURRENT THERAPIES FOR FSGS

The goal of treatment for FSGS is durable remission in proteinuria and preservation of renal function. In proteinuric patients with primary or secondary FSGS (both nephrotic and non-nephrotic), the traditional therapeutic approach consists of blood pressure control and the use of angiotensin-converting enzyme (ACE) inhibitors or angiotensin receptor blockers (176). Current immunosuppressive therapies and conservative management, including inhibitors of the RAAS and sodium-glucose cotransporter, are reviewed elsewhere (177). While this approach remains the mainstay of treatment for those who become non-nephrotic after 6 months of treatment, it is quite rare for severely nephrotic patients to enter partial or complete remission with conservative management alone. Therefore, a more aggressive and prolonged treatment with prednisone or immunosuppressive agents is recommended as a second line of treatment for patients who are persistently nephrotic after a course of conservative therapy or for patients presenting with complications from the nephrotic syndrome (178). However, when the use of high-dose steroids is concerning, other steroid-sparing alternatives such as calcineurin inhibitors or cytotoxic agents such as cyclophosphamide, chlorambucil, and mycophenolate mofetil have proven effective (179). Several lines of experimental evidence indicate that glucocorticoids confer protection to podocytes by attenuating apoptosis and restoring podocyte differentiation markers after injury (180) and that calcineurin inhibitors stabilize the actin cytoskeleton by protecting synaptopodin from degradation (181).

Unfortunately, the treatment of steroid-resistant FSGS presents a greater challenge to nephrologists because patients with this condition not only are unresponsive to steroid therapy but also respond poorly to cytotoxic therapy (182). And, even if patients respond to some extent, another formidable task is to maintain remission and minimize the potential nephrotoxicity associated with such cytotoxic medications (183). Thus, other agents such as abatacept (184, 185), rituximab (186–189), adrenocorticotropic hormone (190), and adalimumab (191), which modulate the immune system by inhibiting inflammation, represent an alternative in patients who do not respond to steroids and other common second-line agents. Clinical trials and outcomes associated with other pro- or anti-inflammatory molecules inducing or preventing glomerular scarring [such as C-C chemokine receptor type 2 (CCR2), Slit2-Roundabout (Robo) signaling pathway, nuclear factor erythroid 2-related factor 2 (Nrf2), and losmapimod, a p38 mitogen-activated protein kinase (MAPK) inhibitor] are thoroughly and comprehensively reviewed by De Vriese et al. (12).

Since proteinuria in FSGS is caused possibly by excessive levels of circulating factors that impair the glomerular filtration barrier, drugs that target the pathogenic circulating permeability factors or treatments that remove them should function as potential FSGS therapeutics. As mentioned above, suPAR is shown to be elevated in 55–85% of patients with primary steroid-resistant FSGS in two large clinical cohorts (45), and subsequent therapy (multiple plasmapheresis sessions) involving reduction in the suPAR levels was associated with improved podocyte health (192). A phase 2 clinical trial with bleselumab, a humanized anti-CD40 monoclonal antibody that neutralizes the circulating pathogenic CD40 autoantibodies in FSGS patients, is underway to prevent the recurrence of disease [National Clinical Trial (NCT) number NCT02921789]. A thorough review on potentially beneficial extracorporeal therapies such as plasma exchange therapy, immunoadsorption, and low-density lipoprotein apheresis (193) for the treatment of FSGS in the pediatric population is provided by Raina et al. (194).

Since fibrosis represents the culmination of glomerular damage in FSGS, a wide range of antifibrotic agents have been proposed as therapeutics. It is widely accepted that transforming growth factor-beta (TGF-β) and its downstream Smad signaling cascade is a key mediator in the pathogenesis of renal fibrosis both in experimental models and in human kidney diseases (195). Direct TGF-β1 inhibitors such as resveratrol, tranilast, and decorin have shown moderate success in animal models but not much in human subjects (196). A combined therapy using a cocktail of p38 MAPK inhibitor and a TGF-β receptor I inhibitor (ALK5I) has been shown to ablate renal injury and reduce the progression of adriamycin (ADR)-induced nephropathy in vivo (197). Administration of TFG-β-neutralizing antibodies such as fresolimumab has also been successful and well tolerated in several types of animal models including diabetic neuropathy (198) and in humans (199, 200). Pirfenidone, an orally active small molecule recognized for its antifibrotic action, showed promise in patients with FSGS (201). Short noncoding RNAs, miRNAs, have been shown to modulate FSGS. For instance, podocyte expression of miRNA-193A was high in patients with FSGS, in contrast with those having MCD. Overexpression of miRNA-193A in mice led to FP effacement, FSGS, and dedifferentiation of podocytes with loss of expression of WT1, podocalyxin, and nephrin; consequently, treatment with an miRNA-193 antagonist resulted in reduced albuminuria and preservation of podocytes (202). Some miRNAs promote (miR-214, miR-21, miR-433) or prevent (miR-30) renal fibrosis independent of TGF-β signaling; thus, their genetic ablation or exogenous expression is able to confer protection (203, 204). MiR-324–3P, which targets prolyl oligopeptidase (POP) and mediates breakdown of thymosin β4 into the antifibrotic tetrapeptide N-acetyl-seryl-aspartyl-lysyl-proline (Ac-SDKP), has been implicated in renal fibrosis (205). ACE has been shown to degrade Ac-SDKP and augment fibrosis. Therefore, ACE inhibitors were able to decrease fibrosis (206).

Molecules that directly work by preserving podocyte structure, function, survival, or viability or that promote podocyte regeneration hold promise as FSGS therapeutics, but they are scarce and understudied. Sparsentan, an endothelin type A (ETA) and AT1R antagonist, was shown to prevent disruption of actin cytoskeleton in experimental FSGS (207), and its protective effects were also evident in a phase 2 study involving patients with FSGS (208–210). Activation of the small GTPase Rac1 has been shown to induce translocation of transient receptor potential canonical-5 (TRPC5) ion channels on the membrane of podocytes, which in turn activates Rac1, eliciting cytoskeletal changes and culminating in podocyte FP effacement and proteinuria (211, 212). A novel TRPC5 channel inhibitor has recently completed phase 1 evaluation in healthy volunteers (NCT03970122), but a phase 2a study (NCT04387448) with FSGS patients has been terminated.

Other therapeutics aim to correct or ameliorate the injurious effects of a genetic mutation on podocyte or GBM structure or function. A recent study demonstrated a reduction of proteinuria in *APOL1*-transgenic mice treated with an *APOL1*-targeted antisense oligonucleotide, providing proof of concept that antisense oligonucleotides can effectively silence the abnormal gene expression with no untoward consequences (213). Additionally, a clinical trial aimed at evaluating the efficacy, safety, and pharmacokinetics of an oral APOL1 inhibitor, VX-147, in patients with an FSGS lesion and *APOL1* G1/G1, G2/G2, or G1/G2 genotypes is ongoing (NCT04340362).

A detailed review summarizes recent advances made in FSGS treatment (including current clinical trials) and describes novel paradigms and emerging therapeutics (214). Despite decades of painstaking research and numerous clinical trials, no therapeutic target has been identified that is applicable to all patients, a situation attributable to the lack of well-defined animal models representing different FSGS subtypes. As FSGS is a heterogeneous disease, it seems likely that a multipronged approach will be needed to develop highly effective treatments (215).

## 7. CONCLUSION

**Figure 2:**
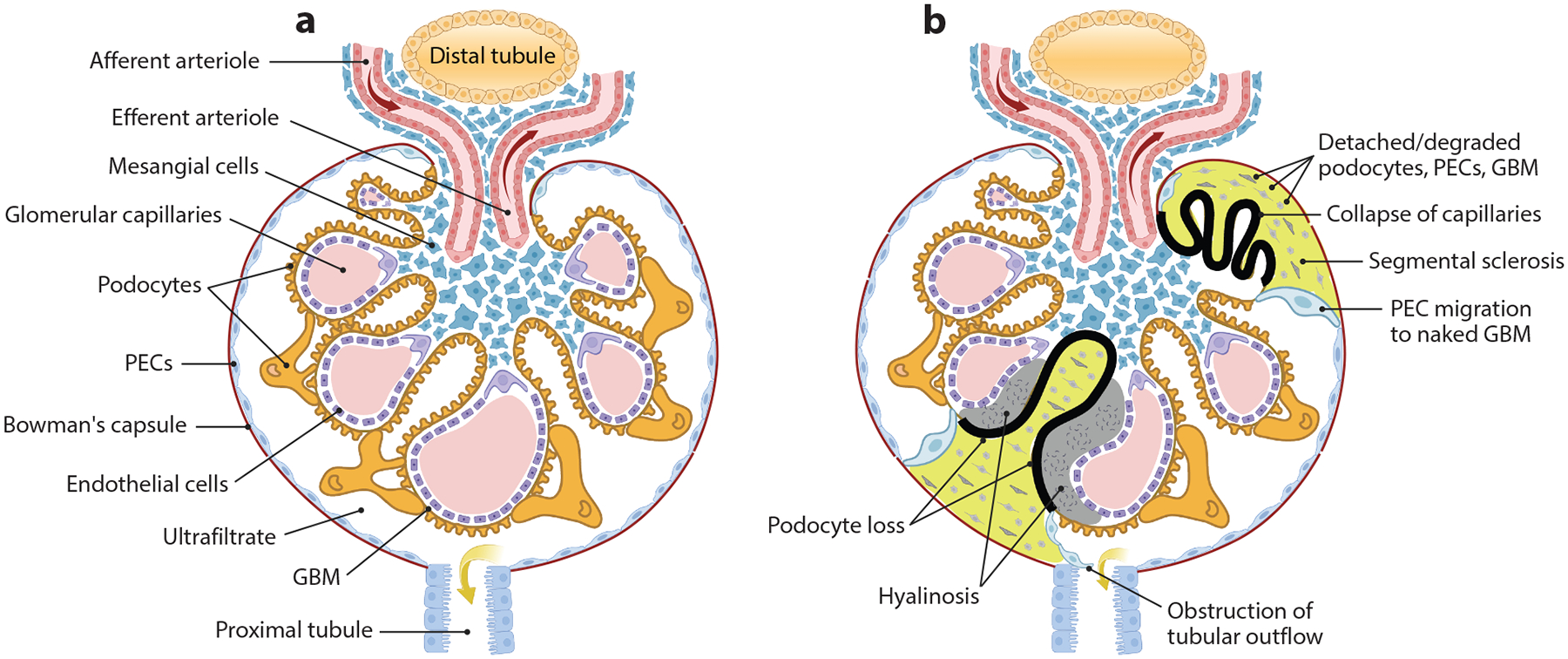
Common glomerular histological changes in FSGS. (*a*) A normal glomerulus with a tuft of capillaries and surrounding proximal and distal convoluted tubules. (*b*) The partially sclerosed glomeruli in FSGS. The condition is characterized by the effacement, detachment, and death of podocytes, resulting in an area of denuded GBM and extensive deposits composed of the remains of damaged podocytes, PECs, and GBM components. FSGS lesions are associated with adhesion of PECs, which are originally located along the Bowman’s capsule, to the glomerular tuft. In some cases, the lesions form hyalinosis (prominent serum protein casts), causing obliteration of glomerular capillaries. Additionally, activated PECs may migrate toward the tubular orifice and obstruct the urine flow. Abbreviations: FSGS, focal segmental glomerulosclerosis; GBM, glomerular basement membrane; PEC, parietal epithelial cell. Figure adapted from images created with BioRender.com.

FSGS is a rare glomerular disease that causes scarring in parts of some glomeruli, the filters of the kidney (Figure 2). Identification of glomerular lesions can be challenging due to their pathological heterogeneity with respect to their position at the glomerular vascular and tubular poles. Therefore, various computer-aided morphometric analysis techniques were developed to provide a robust framework to characterize the lesions, to identify their subtypes (on the basis of the appearance), and even to associate these lesions with the subcategories of FSGS, including primary, secondary, and genetic. The specific cause of primary FSGS is often unknown, but certain circulating factors have been implicated to target podocytes, leading to FP effacement, increased permeability through the damaged filtration barrier, and massive proteinuria, which are hallmarks of FSGS. The most investigated circulating factor is suPAR; research to disclose how it mediates primary FSGS is ongoing. Patients with primary FSGS can experience a rapid deterioration in their kidney function, whereas kidney failure often develops at a slower pace in patients with secondary FSGS. On the other hand, signs and symptoms of genetic forms of FSGS are extremely variable among patients, from mild disease with few to no symptoms to severe disease with nephrotic syndrome. Defining the particular type of FSGS is important for both prognostic and therapeutic purposes, as primary FSGS often requires immunomodulatory treatment whereas management of secondary FSGS is directed toward the reduction of intraglomerular hypertension using RAAS blockade.

In summary, there is an unmet need for innovative treatment strategies for FSGS patients that improve outcomes and reduce the progression to ESRD. We envision that advances in translational research will increase our knowledge on the pathogenesis of FSGS and eventually enable the development of safe and efficacious therapies.

## Figures and Tables

**Table 1:** Columbia classification for FSGS

Histologic variant	Features
Cellular	Characterized by the presence of at least one glomerulus with segmental (involving at least 25% of the tuft) endocapillary hypercellularity and causing occlusion of the capillary lumen (139). A stepwise diagnostic process is recommended for the cellular variant, which requires the exclusion of collapsing and tip variants.
Collapsing	Characterized by the presence of at least one glomerulus with segmental and global collapse (140). Podocytes undergo phenotypic changes (e.g., hypertrophy is common and is usually accompanied by cytoplasmic hyaline droplets) and do not have characteristics of mature podocytes such as proliferation (141) and dedifferentiation (142). This variant typically associates with the most rapidly progressive presentations when compared with other variants of FSGS.
Tip	The segmental glomerular scarring represents the outer (i.e., peripheral) 25% of the glomerular tuft and occurs at the tubular pole (i.e., glomerular “tip” adjacent to the beginning of the proximal tubule). Podocytes demonstrate a confluent morphology at the tubular lumen, along with parietal and tubular cells. The definite diagnosis requires the absence of perihilar or collapsing lesions. It is a clinically different variant, where the symptoms often appear suddenly, steroid responsiveness is high (with a chance of complete remission), and chronic renal damage is less severe (143).
Perihilar	Well differentiated with the presence of at least one glomerulus with hyalinosis in more than half of the sclerotic glomeruli in the perihilar area, where the blood vessels enter and exit. A recent study provided clues for the involvement of PECs in the development of perihilar lesions in the Munich–Wistar rat model of FSGS (144).
FSGS-NOS	None of the specific pathological conditions listed above occur. This represents the most common subtype of FSGS.

Abbreviations: FSGS, focal segmental glomerulosclerosis; NOS, not otherwise specified; PEC, parietal epithelial cell.
